# Cholinergic Dynamics in the Septo-hippocampal System Provide Phasic Multiplexed Signals for Spatial Novelty and Correlate with Behavioral States

**DOI:** 10.1523/JNEUROSCI.0133-25.2025

**Published:** 2025-09-10

**Authors:** Fatemeh Farokhi Moghadam, Blanca E. Gutiérrez-Guzmán, Xihui Zheng, Mina Parsa, Lojy M. Hozyen, Holger Dannenberg

**Affiliations:** ^1^Department of Bioengineering, George Mason University, Fairfax, Virginia 22030; ^2^Interdisciplinary Program in Neuroscience, George Mason University, Fairfax, Virginia 22030

**Keywords:** acetylcholine, encoding and retrieval, fiber photometry, hippocampal formation, medial septum, novel object location

## Abstract

In the hippocampal formation, cholinergic modulation from the medial septum/diagonal band of Broca is known to correlate with the speed of an animal's movements at subsecond timescales and also supports spatial memory formation. Yet, the extent to which subsecond cholinergic dynamics, if at all, align with transient behavioral and cognitive states supporting the encoding of novel spatial information remains unknown. In this study, we used fiber photometry to record the temporal dynamics in the population activity of septo-hippocampal cholinergic neurons at subsecond resolution during a hippocampus-dependent object location memory task using ChAT-Cre mice of both sexes. Using a linear mixed-effects model, we quantified the extent to which cholinergic dynamics were explained by changes in movement speed; behavioral states such as locomotion, grooming, and rearing; and hippocampus-dependent cognitive states such as recognizing a novel location of a familiar object. The data show that cholinergic dynamics contain a multiplexed code of fast and slow signals (1) coding for the logarithm of movement speed at subsecond timescales, (2) providing a phasic spatial novelty signal during the brief periods of exploring a novel object location, and (3) coding for recency of environmental change at a seconds-long timescale. Furthermore, behavioral event-related phasic cholinergic activity demonstrates that fast cholinergic transients correlate with a switch in cognitive and behavioral states. These findings enhance understanding of the mechanisms by which cholinergic modulation contributes to the coding of movement speed and encoding of novel spatial information.

## Significance Statement

Acetylcholine (ACh) is well known as a neuromodulator of cognitive functions and behavior, and computational models suggest an important role in the encoding of new memories. However, whether cholinergic dynamics are fast enough to serve as a spatial novelty signal is unknown. Here, we demonstrate that cholinergic signaling in the septo-hippocampal circuitry of mice exhibits multiple timescales of activity, where fast signals reflect the detection of novel object locations, encode the logarithm of movement speed, and correlate with behavioral state transitions. At longer timescales, cholinergic transients encode recency of environmental change. These findings provide important insights into the mechanisms by which ACh contributes to encoding and retrieval dynamics and the acquisition of spatial memories during exploratory behavior and memory-guided navigation.

## Introduction

Cognitive map-based navigation requires the encoding, storage, and recall of spatial information to navigate environments effectively (Schiller et al., 2015). This process integrates sensory inputs, motor planning, and memory, relying on a mental representation of both current and future locations (Hinman et al., 2018). The hippocampal formation plays a central role in spatial navigation by supporting the formation and retrieval of spatial memories (Eichenbaum, 2017). Cholinergic projections to the hippocampal formation arise from cholinergic neurons in the medial septum/diagonal band of Broca (MSDB; Dutar et al., 1995). Changes in cholinergic modulation by medial septal projection neurons can cause changes in brain and behavioral states (Mesulam et al., 1983; Alonso and Köhler, 1984; Rye et al., 1984; Dannenberg et al., 2017; Hinman et al., 2018). In the hippocampus, optogenetic stimulation of cholinergic projection neurons induces theta and gamma power in anesthetized mice (Dannenberg et al., 2015) and enhances theta rhythmic activity while suppressing sharp wave–ripples (SPW–Rs) in behaving mice (Vandecasteele et al., 2014). These findings support the influential hypothesis that acetylcholine (ACh) enhances processing of feedforward sensory input to the cortex to promote the encoding of novel memories while suppressing retrieval of previously stored memories via feedback excitation (Hasselmo et al., 1995; Hasselmo, 2006). Conversely, during quiet wakefulness and non-REM sleep, cholinergic tone is reduced, allowing feedback excitation to dominate during SPW–Rs serving memory consolidation and action planning (Rogers and Kesner, 2003; Hasselmo and McGaughy, 2004; Hasselmo, 2006; Barry et al., 2012; Fernández-Ruiz et al., 2019; Zhang et al., 2024, 2021).

While neuromodulation by ACh in the hippocampal formation has been linked to the encoding of spatial memories by modulating network dynamics, synaptic plasticity, and neuronal excitability (Blokland et al., 1992; Ohno et al., 1993; Stancampiano et al., 1999; Hasselmo, 2006; Hasselmo and McGaughy, 2004; Dannenberg et al., 2017; Hasselmo et al., 2017; Záborszky et al., 2018), recent mouse studies suggest that cholinergic signals may provide a code for movement speed and promote processing of sensory information associated with exploratory behavior, as opposed to modulating memory function (Dannenberg et al., 2015; Kopsick et al., 2022; Cassity et al., 2024). In addition, cholinergic modulation has been suggested to provide a novelty signal to distinguish between familiar and novel spatial information (Gómez-Ocádiz et al., 2022). Yet, how cholinergic dynamics in the septo-hippocampal system can support those multiple cognitive and sensory–motor functions is largely unknown. While a multiplexed combination of fast and slow cholinergic signals has been demonstrated in the auditory cortex (Zhu et al., 2023), it remains unclear whether cholinergic modulation in the septo-hippocampal system can provide a multiplexed combination of fast and slow signals. Concretely, whether cholinergic dynamics are fast enough to provide novelty signals that can support the encoding of novel spatial information during active exploratory behavior remains largely unknown.

In this study, we used fiber photometry to record the population activity of cholinergic neurons in the MSDB at subsecond timescales. These recordings were performed using ChAT-Cre mice engaged in an object location memory (ObLoM) task. Mice were video-tracked to quantify movement speed and behavioral states. The results show that cholinergic activity in the septo-hippocampal circuitry exhibits multiplexed coding: fast cholinergic dynamics provide a code for movement speed and transient spatial novelty signals during the brief moments of object exploration at novel but not familiar locations. In contrast, slow changes in cholinergic activity are associated with the recency of environmental change. These data contribute to our understanding of the mechanisms by which cholinergic modulation contributes to the encoding of spatial memories, including novel object–place associations.

## Materials and Methods

### Animals

We used a total of six transgenic heterozygous mice (Table 1) expressing Cre-recombinase under the control of the choline acetyltransferase (ChAT) promoter (ChAT-IRES-Cre) at the age of 3–8 months. Breeder pairs were purchased from the Jackson Laboratory, and mice were bred in-house (C57BL/6J X B6;129S6Chat^TM2(cre)Lowl^/J, 3 mice; C57BL/6J X B6;129S6Chat^TM1(cre)Lowl^/MwarJ, 4 mice). This study includes data from three mice which were also used to collect additional data for a previously published study (Kopsick et al., 2022). Prior to surgery, mice were housed in Plexiglas cages with their siblings. Postsurgery, they were individually housed in extended-height cages on a 12 h reversed light/dark cycle with food and water available *ad libitum*. The cages included a spherical treadmill for enrichment, physical exercise, and stress relief. All mice were handled and habituated to the experimenter and testing room prior to the start of experiments. Experimental procedures for newly acquired data adhered to the regulations outlined in the Guide for the Care and Use of Laboratory Animals by the National Research Council and approved by the Institutional Animal Care and Use Committee of George Mason University (Protocol No.: 0501, approved on January 24, 2022).

**Table 1.** Information on mice used in experiments

Mouse ID	Gender	Age (months)	Number of ObLoM tasks	Genotype (heterozygous)	GCaMP version	Performance of ObLoM task (days after surgery)	Perfusion for histology (days after surgery)
#1	Male	8	1	Chat^TM1(cre)Lowl^/MwarJ	jGCaMP8m	12	18
#2	Male	6	2	Chat^TM1(cre)Lowl^/MwarJ	jGCaMP8s	15, 21	44
#3	Male	4	1	Chat^TM1(cre)Lowl^/MwarJ	jGCaMP8s	12	23
#611915^a^	Female	3–6	2	Chat^TM2(cre)Lowl^/J	jGCaMP7s	10, 14	–
#611916^a^	Female	3–6	2	Chat^TM2(cre)Lowl^/J	jGCaMP7s	11, 16	–
#611926^a^	Male	3–6	2	Chat^TM2(cre)Lowl^/J	jGCaMP7s	11, 15	–
#4	Male	4	–	Chat^TM1(cre)Lowl^/MwarJ	jGCaMP8s	–	13

ObLoM task, Object location memory task.

a Data from this mouse were acquired in the laboratory of Dr. Hasselmo at Boston University.

### Surgery

Anesthesia was induced with 4% isoflurane in a gas chamber and maintained at 1–2% (mixed with 100% oxygen) via the nose cone on the stereotaxic instrument. The body temperature was kept steady and monitored throughout the surgery using a heating pad and homeothermic monitoring system (36–37°C). Preoperative care included administering atropine (0.1 mg/kg, s.c.), ketoprofen (5 mg/kg, s.c.), enrofloxacin (7.5 mg/kg, s.c.), and local anesthetic (lidocaine 0.5%, 5 mg/kg) underneath the scalp before making the surgical incision.

#### Virus injection

Craniotomy was performed for a recombinant adeno-associated virus injection of calcium indicators from the GCaMP family (jGCaMP7s, jGCaMP8s, or jGCaMP8m; Table 1) and for the chronic implantation of an optical fiber above the MSDB. Both procedures use the same coordinates from the Paxinos and Franklin atlas (Paxinos and Franklin, 2019): 1.05 mm anterior from the bregma and 0.7 mm lateral to the bregma. The injection needle (Table 2) was lowered into the MSDB at an 8° polar angle and a −90° azimuth angle, and total volume of 1,000 nl of virus solution was injected at a rate of 100 nl/min at two ventral sites, −4.8 and −4.4 mm below the skull surface (500 nl at each), using an injection pump (Table 2). The needle for virus injection was left in place for 10 min after each injection to ensure diffusion and prevent backflow of the virus solution.

**Table 2.** Materials and resources

Material	Specifications
Injection needle	NanoFil Needle NF34BV, 34G, beveled, World Precision Instruments (WPI)
Injection pump	UMP3T-1; MICRO2T SMARTouch controller, WPI
Implantable optical fiber	MFC_200/250-0.66_7mm_ZF1.25(G)_FLT; Doric Lenses
Fiber photometry system	RZ10X, Tucker-Davis Technologies
Mono fiber-optic patch cord	MFP_200/230/900-0.57_2.5m_FC-MF1.25(F)_LAF, Doric Lenses
Rotary joint	FRJ_1 × 1_FC-FC, Doric lenses
DPBS	Catalog #14040133, Thermo Fisher Scientific
Vibratome	VT1000 S, Leica
Goat anti-ChAT affinity purified polyclonal antibody	Catalog #AB 144P, Merck Millipore
Triton X-100 detergent	X100-100ML, Sigma-Aldrich
Cy3-conjugated donkey anti-goat IgG polyclonal antibody	Catalog #AP180C, Merck Millipore
Aqua-Poly/Mount	#18606-20, Polysciences
Confocal laser scanning microscope	LSM 800, Carl Zeiss

#### Implantation of optical fiber

An optical fiber (total diameter, 250 µm; 200 µm core; N.A. 0.66; Table 2) was implanted at the same coordinates and angle used for the virus injection. The fiber was lowered 4.2 mm from the skull surface and secured to the animal's skull with black dental cement. Four stainless steel anchoring screws were implanted into the skull to reinforce the attachment of the dental cement and optical fiber. Care was taken that the anchoring screws did not protrude into the brain tissue.

#### Postsurgical care

Mice received ketoprofen (5 mg/kg, s.c.) and enrofloxacin (7.5 mg/kg, s.c.) for 2 d after surgery. They were given 1 week for full recovery before the start of experiments.

### Fiber photometry

Newly acquired data were collected with a fiber photometry system (Table 2) [Tucker-Davis Technologies (TDT); RZ10X], which is similar to the previous custom-made system used in Kopsick et al. (2022) but provided several improvements with respect to the signal-to-noise ratio, including a more stable nonmagnetic connection to the implanted optical fiber to reduce movement noise. The TDT system used lock-in amplification, configured with a 465 nm LED driver modulated at 211 Hz for Ca^2+^-dependent excitation of GCaMP and a 405 nm UV LED driver modulated at 531 Hz for excitation of GCaMP at its isosbestic point. Laser light was transmitted into the brain using fiber-optic patch cords connected through a rotary joint (Table 2). The power of the laser entering the implanted optical fiber was assessed both before and after each recording session and adjusted to deliver ∼40 µW of laser power into the MSDB. The data were demodulated with a sixth-order low–pass filter set to 6 Hz. LED currents were adjusted to return a voltage between 5 and 10 mV for each signal, offset by 5 mA. Data were acquired at a sampling rate of 610 Hz. The TDT Synapse software was used for data acquisition.

### Behavioral tests and video-tracking

Data were acquired from animals performing an ObLoM, which relies on spontaneous behavior. The behavioral tests were performed in a dimly lit room dedicated for behavioral experiments with no windows to minimize external stimuli.

#### Arena and objects

The recording arena consisted of an open-field box made of black acrylic, measuring 40 × 40 cm^2^ with 30 cm high walls. A visual cue card (a white triangle) was placed on one wall, the same wall across all experiments. Two identical cylindrical objects (50 ml conical tubes with a base diameter of 3.4 cm) were placed 10 cm away from two adjacent or nonadjacent corners of the arena in the sample and test phases, respectively.

#### ObLoM task

We conducted the ObLoM task as described previously (Schlesiger et al., 2021). The task consisted of two phases, a sample session and a test session, each lasting 15 min and separated by a 1 h delay during which the mice were returned to their home cages. To prevent olfactory interference and potential object bias, the maze and objects were cleaned with 70% isopropanol between sessions. In four out of six mice (Table 1), the ObLoM task was repeated in a counterbalanced design, with the stationary and nonstationary objects reversed to control for potential location preferences. The interval between tasks for each mouse ranged from 4 to 6 d (mean, 4.75; SD, 0.95). Prior to the ObLoM task, mice were habituated to the experimenter, the recording room, and the maze. During this habituation period, they were allowed to explore *ad libitum* the open-field box for at least 20 min per day over the course of 5 d.

#### Data acquisition

Fiber photometry data were acquired in continuous recordings during all phases of the memory task (sample, delay period, and test), within a time window of 10–21 d after the virus injection.

#### Video-tracking

Mice were video-tracked using a camera ceiling-mounted above the arena. The frame rate ranged from 10 to 30 fps. All recordings were subsequently resampled via Fourier-based interpolation to 30 Hz for consistent comparison. The camera and the fiber photometry system were synchronized using TTL pulses.

### Histology

#### Tissue preparation

Mice were deeply anesthetized with isoflurane and transcardially perfused with Dulbecco's phosphate-buffered saline (DPBS; Table 2) containing 0.9% calcium chloride, followed by 10% buffered formalin. Mice were decapitated postperfusion, and the heads were stored in formalin for 24–48 h at 4°C for further tissue fixing. Brains were then extracted and stored in DPBS at 4°C.

#### Sectioning and staining

The 30 µm coronal slices of the medial septum were collected in DPBS using a vibratome (Table 2). Immunohistochemical staining for ChAT in the MSDB was performed as described previously (Kopsick et al., 2022). Slices were washed three times for 15 min with DPBS containing 0.9% calcium chloride, then incubated with a goat anti-ChAT affinity purified polyclonal antibody (Table 2), and diluted 1:200 with 0.3% Triton X-100-DPBS (Table 2) for 2 d at 4°C. For the secondary antibody incubation, after washing three times with DPBS, slices were incubated for 2 h at room temperature with a Cy3-conjugated donkey anti-goat IgG polyclonal antibody (Table 2), diluted 1:200. Finally, slices were washed three more times, mounted on glass slides with Aqua-Poly/Mount (Table 2).

#### Imaging

Slides were imaged using a confocal fluorescent microscope (Table 2) with a suitable objective (10 and 20×). Optical fiber placements were histologically verified by visualizing fiber tracks using the mouse atlas (Paxinos and Franklin, 2019) and checking the alignment of GCaMP expression with regions containing ChAT-positive neurons.

### Data analysis

#### Fiber photometry signal

Signal processing was performed as reported previously (Kopsick et al., 2022). Briefly, we accounted for photobleaching and motion artifacts in the fiber photometry signal by subtracting an adjusted control signal from the calcium signal. To compute the adjusted control signal, we first subtracted the isosbestic control signal from the calcium signal, fitted a second-degree curve to the result of that subtraction, and added the fitted curve back to the isosbestic control signal. In a second step, we scaled the adjusted control signal's amplitude so that the correlation with the calcium signal was maximal. Specifically, we created an optimization task to determine the values of two parameters, α and β, minimizing the expression $\Sigma (s\;-\;(c\;*\;\alpha \;+\;\beta ){)}^{2}$, where s represents the main signal and c represents the adjusted control signal. Lastly, we computed ΔF/F as (s−(c*α+β)/(c*α+β)).

#### Calculation of movement speed using markerless pose estimation

We utilized DeepLabCut (Mathis et al., 2018), a deep learning tool, for markerless pose estimation of mice's body parts, including the neck and tail base. To estimate the animal's movement speed, we tracked the neck position in the maze.

#### Speed tuning across different time scales

To determine the timescale at which changes in movement speed and cholinergic activity aligned best, we calculated the Pearson's correlation coefficient between the logarithm of the animal's movement speed and the cholinergic signal measured as ΔF/F using logarithmically increasing smoothing window sizes ranging from 0.25 to 256 s (Dannenberg et al., 2019).

#### Classification of behavioral profiles

We utilized DeepEthogram (Bohnslav et al., 2021) to characterize behavioral profiles in mice during experimental sessions. For our study, four different experimenters manually labeled a randomly selected 2–3 min window from each video based on a predefined list of behaviors to create the training dataset. We then trained DeepEthogram's algorithm to label the remaining video portions. The output provided a list of frame numbers with binary indicators (1 or 0) indicating the presence or absence of each behavior. We focused on classifying locomotion, rearing, grooming, and object exploration. Locomotion referred to any movement that resulted in a change in the animal's location in space. Rearing was defined as any instance of the mice standing on their hind legs, either supported or unsupported by a wall or an object. Grooming includes any self-maintenance behavior, such as licking fur, scratching, or face washing. Object exploration included any interaction with an object where it was clear that the mouse's attention was focused on the object, such as sniffing the object, exploring the immediate surrounding of the object, stretched-attend behavior toward the object, or rearing supported by the object. In cases where the animal's behavior was ambiguous, such as when it was unclear whether the mouse was sniffing the ground, standing still, grooming, sniffing walls or corners, or transitioning between behaviors, those segments were labeled as background. To determine whether object exploration occurred at the stationary or nonstationary object, we used positional data extracted from DeepLabCut. If object exploration occurred while the animal's nose was within a 10 cm radius around the center of the stationary or nonstationary object, we classified the behavior as exploring the stationary or nonstationary object, respectively. Finally, one experimenter confirmed all labels and corrected errors made by DeepLabCut and DeepEthogram to improve the accuracy of the exact start and end times of each behavior.

#### Discrimination index

To quantify the animal's performance in the ObLoM task, i.e., the ability to distinguish between a familiar and novel spatial location of objects, we used the discrimination index (DI). The DI index was calculated as follows:

$$\mathrm{Discrimination}\;\mathrm{index}=\frac{\mathrm{Time}\;\mathrm{spent}\;\mathrm{on}\;\mathrm{Non}-\mathrm{stationary}\;\mathrm{Object}-\mathrm{Time}\;\mathrm{Spent}\;\mathrm{on}\;\mathrm{Stationary}\;\mathrm{Object}}{\mathrm{Time}\;\mathrm{spent}\;\mathrm{on}\;\mathrm{Non}-\mathrm{stationary}\;\mathrm{Object}+\mathrm{Time}\;\mathrm{Spent}\;\mathrm{on}\;\mathrm{Stationary}\;\mathrm{Object}}.\;$$

#### Linear mixed-effects model

To differentiate between the individual effects of each behavior on cholinergic activity during sample versus test sessions, we employed a linear mixed-effects model (LMM) with the following predictors: the logarithm of movement speed, rearing, grooming, exploring stationary object, and exploring nonstationary object. All behavioral predictors were modeled with their interaction terms with task phase (sample vs test). Given the high collinearity between movement speed and locomotion, only movement speed was included as a continuous, quantitative representation of locomotor behavior. All cholinergic signals were downsampled to 30 Hz to match the video frame rate. Before fitting the model, both cholinergic and speed signals were smoothed using a 0.5 s moving average window. Data from all sessions were aggregated into a single model (*n* = 20; 10 sample and 10 test sessions). We accounted for within- and between-subject variability by including random effects for session ID, mouse ID, and ObLoM ID nested within mouse ID (the ObLoM task was repeated in four out of six mice). Random effects were applied to both intercepts and slopes. Random effects and residual errors were assumed to follow a normal distribution with a mean of zero. The hierarchical modeling approach effectively captured the nested structure of the dataset while preserving session-specific signal baselines, amplitudes, and behavioral dynamics. Such considerations are critical in the analysis of fiber photometry data, where GCaMP expression levels, the signal-to-noise ratio, and motion artifacts can vary substantially across sessions. MATLAB scripts used for model fitting and the generation of the statistical results tables are available on GitHub to ensure transparency and reproducibility.

#### Event-triggered cholinergic activity

For each behavior, we extracted the onset and offset times and analyzed the *z*-score of cholinergic activity measured as ΔF/F between the onset and offset as well as in the 5 s before and the 5 s after the onset and offset of the behavior, respectively. We removed any short bouts (<2 s) and excluded bouts if the same behavior occurred within 4 s before the onset or after the offset of the bout. To average event-triggered cholinergic activity across events, we standardized the data points between onset and offset by linearly time-warping the cholinergic activity to a uniform length. This approach allowed us to compute an event-triggered temporal profile of cholinergic dynamics for each behavior.

#### Proportion of total behaviors

Based on the definition of different behavioral states, object exploration could overlap in time with rearing or locomotion. To quantify this overlap, we computed the proportion of total behaviors by computing the ratio between the occurrence of an individual behavior at a given time and the sum of all behaviors occurring at that time. For this analysis, behavioral bouts from all sessions were included.

#### Quantification of event-triggered temporal profile of cholinergic activity

To determine the time points when cholinergic activity rises and falls relative to the onset and offset of each behavioral event, we first computed a threshold indicating a meaningful change in activity. We defined the baseline as the average activity 5 s before the onset (or after the offset) and computed the threshold as the sum of the baseline and 98% of the difference between the average activity during the behavior and the baseline. We then identified the time points at which the cholinergic signal crossed this threshold within a window of 3 s before and 1 s after the onset (or 3 s after and 1 s before the offset). For each behavioral bout, the median of these threshold-crossing time points was taken as the estimate of cholinergic response timing. To compare the timing between observed and speed-predicted cholinergic activity, we analyzed the distributions of these rise and decay estimates across behavioral bouts and reported their median differences.

#### Nonparametric cluster-based permutation test

To statistically assess temporal differences between observed and speed-predicted cholinergic signals, we employed a nonparametric cluster-based permutation test as described in detail by Maris and Oostenveld (2007). Briefly, for each time point, we performed a paired *t* test, yielding a time-series of *t* values. Clusters were defined as contiguous time points where the *t* values exceeded a significance threshold corresponding to a significance level α = 0.05 (for a two-sided test). For each cluster, we computed two cluster-level statistics: cluster mass (the sum of *t* values within the cluster) and cluster length (the number of contiguous significant time points). To assess the statistical significance of these clusters, we ran 500 and 1,000 permutations to analyze effects of recency of environmental change and behavioral bouts, respectively, in which the condition labels (observed vs predicted) were randomly shuffled. For each permutation, the cluster extraction procedure was repeated, and the maximum cluster mass and length were recorded. This resulted in null distributions to perform cluster-level statistics. Clusters were considered statistically significant if their absolute cluster mass or length exceeded the 95th percentile of the null distributions, corresponding to a Monte Carlo *p* < 0.05. This method allowed us to identify time periods where the signals diverged significantly while controlling the family-wise error rate. Additionally, we report the relative lengths of significant clusters as a percentage of duration.

### Statistical analysis

Data analysis and statistical tests were conducted using custom-written Python and MATLAB scripts and DATAtab (DATAtab, n.d.), with all codes and processed data accessible via the GitHub repository (https://github.com/dannenberglab/Cholinergic-dynamics-in-spatial-exploration). Cholinergic activity and movement speed were smoothed using a 0.5 s moving average window. This method effectively reduced high-frequency noise while preserving the behaviorally relevant temporal dynamics of the signal. Correlations between time-series data were assessed using Pearson's correlation coefficient. Predicted cholinergic activity based on movement speed was calculated using a linear regression model on the *z*-scored observed cholinergic activity. An LMM was applied using built-in MATLAB functions to evaluate the effects of behavioral signals and movement speed on cholinergic activity. Post hoc power analysis was performed using G*Power (Faul et al., 2009, 2007) based on effect size estimates and variability parameters derived from the LMM results. The Kolmogorov–Smirnov test and visual inspection of *Q*–*Q* plots were used to assess whether the distributions of data were approximately normal; parametric and nonparametric tests were applied accordingly. Statistical tests were two-tailed, and significance was determined using an α level of 0.05.

## Results

### Cholinergic activity is robustly correlated to the logarithm of the animal's movement speed

**Figure 1.**
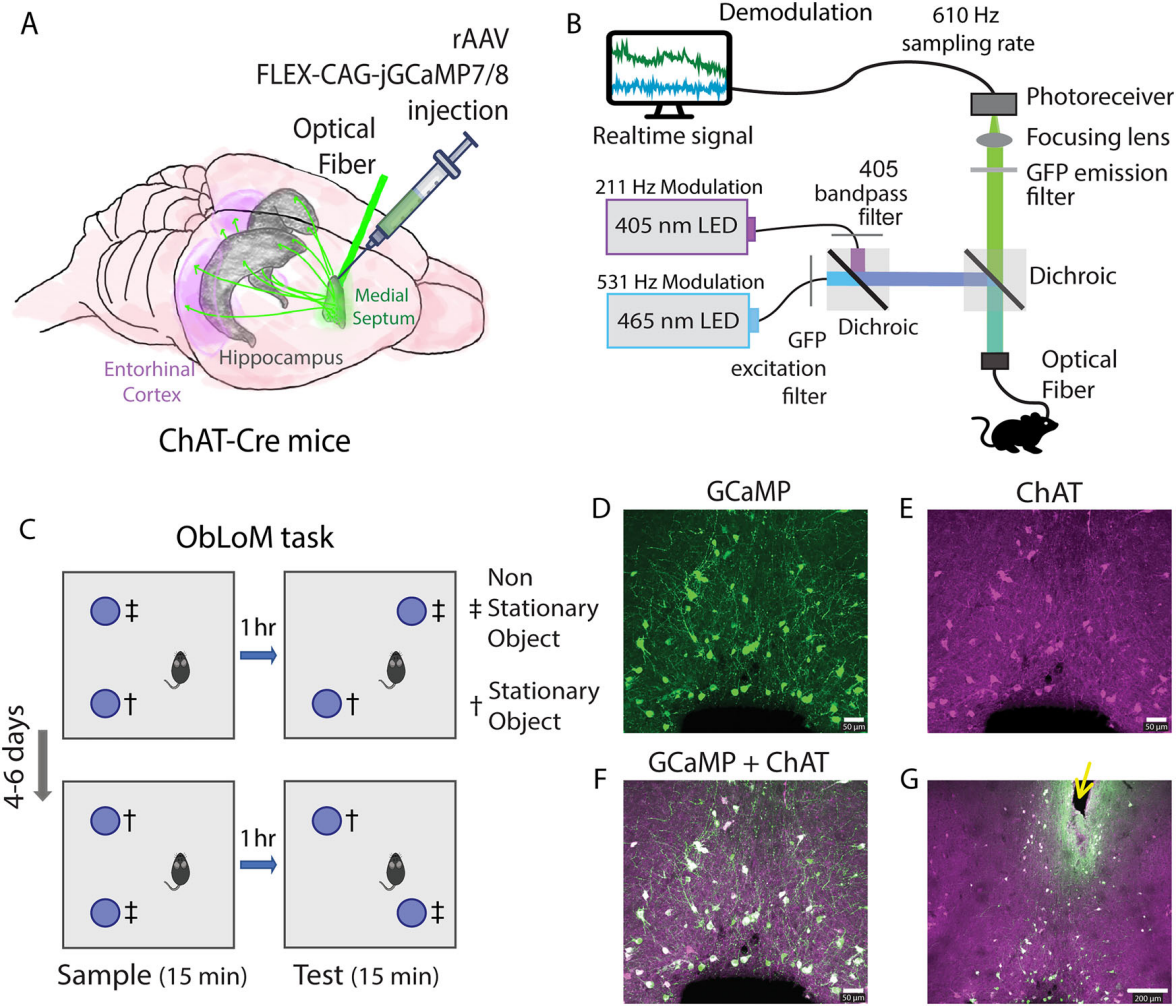
Experimental design. ***A***, Schematic drawing of the surgical approach taken to perform fiber photometry of cholinergic septo-hippocampal projection neurons using jGCaMP7/8 in *ad libitum* behaving ChAT-Cre mice. ***B***, Schematic drawing of the fiber photometry system. ***C***, Design of the ObLoM task. During the sample phase (15 min), mice explored two identical objects in a 40 × 40 cm^2^ square arena with 30 cm high walls. During the test phase (15 min), one of the two objects was moved to a novel location (nonstationary object). The sample and test phases were separated by a 1 h delay phase, during which the mouse was returned to its home cage (6 mice). The ObLoM task was repeated in a counterbalanced design after 4–6 d (4 mice). ***D–G***, Immunohistological verification of the optical fiber track position and cell type-specific expression of jGCaMP7/8 in cholinergic neurons of the MSDB (Mouse ID #4). (***D***) The green color indicates jGCaMP7/8 fluorescence; (***E***) the magenta color indicates immunolabeling of ChAT, a marker for cholinergic neurons; (***F***) the white color indicates colocalization of jGCaMP7/8 fluorescence and ChAT immunostaining (Scale bar, 50 µm); (***G***) histological confirmation of the fiber track position (yellow arrow points to the tissue displaced by the implanted optical fiber) within the MSDB (Scale bar, 200 µm).

First, we addressed whether cholinergic dynamics in the septo-hippocampal circuitry enable multiplexed coding of movement speed. Toward that goal, we measured the population activity of septo-hippocampal cholinergic projection neurons using fiber photometry in behaving mice performing a hippocampus-dependent ObLoM task (Fig. 1*A*,*B*). The ObLoM task consisted of a sample and a test session. In four out of six mice, the ObLoM task was repeated once after 4–6 d, with the other object being moved to a novel location. The complete dataset therefore includes 10 sample and 10 test sessions (Table 1; Fig. 1*C*). Immunohistological data from coronal brain slices of the MSDB showed colocalization of GCaMP-positive neurons and ChAT-immunolabeled neurons, confirming that GCaMP expression was largely confined to cholinergic neurons in the MSDB (Fig. 1*D*–*G*).

**Figure 2.**
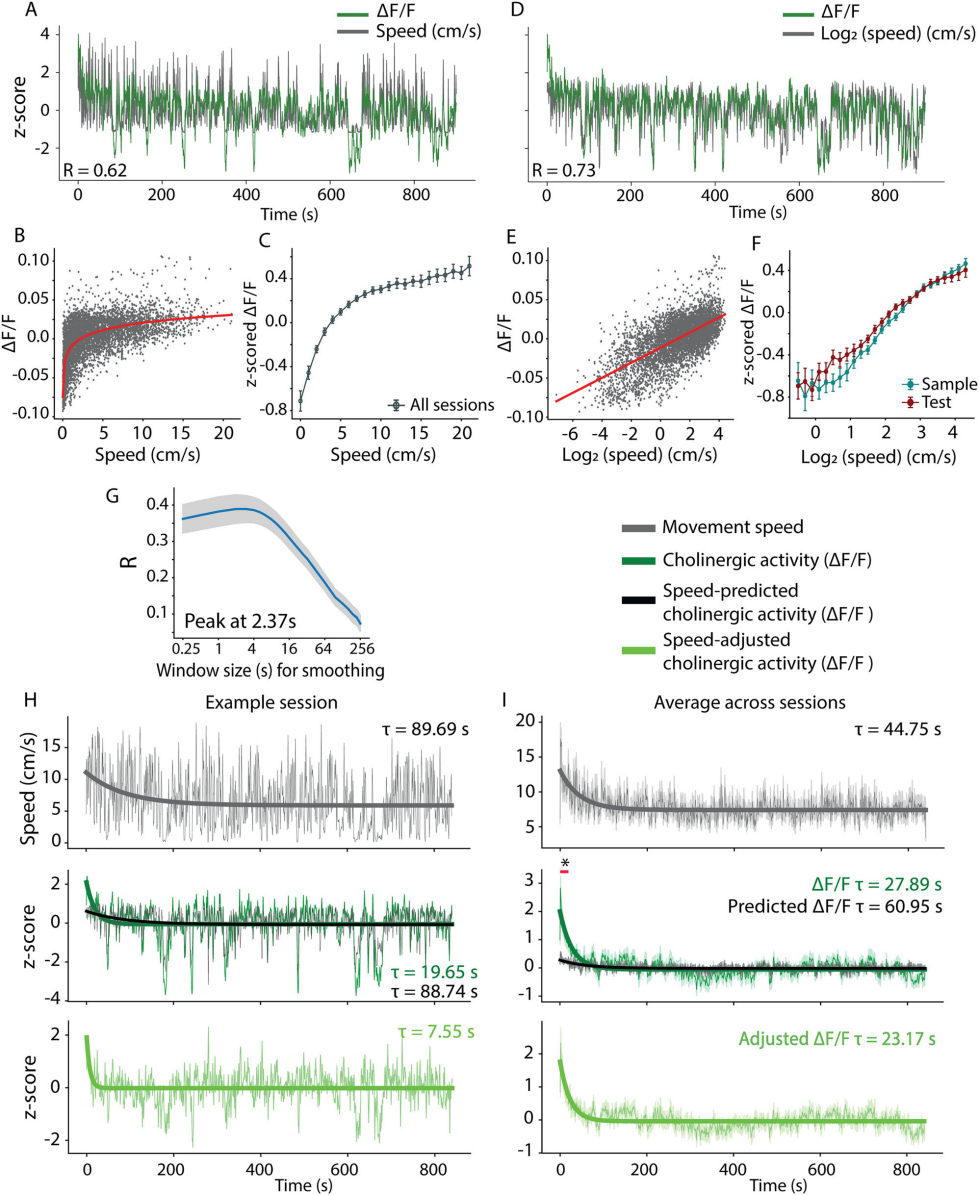
Cholinergic activity is linearly correlated to the logarithm of the animal's movement speed during an ObLoM task. ***A***, Correlation between cholinergic activity, quantified as Δ*F*/*F*, and the animal's movement speed, for one example session recorded during the test phase. Signals are smoothed with a 1 s window; R, Pearson's correlation coefficient. ***B***, Scatter plot of cholinergic activity, quantified as Δ*F*/*F*, as a function of the animal's movement speed for an example session, with an exponential function fitted to the data (red). ***C***, *Z*-scores of Δ*F*/*F* of cholinergic activity across all sessions as a function of the animal's movement speed (20 sessions). Speed is binned with a bin width of 1 cm/s. Data shown as mean ± SEM. ***D***, Same data as in ***A*** but using the logarithm of the animal's movement speed. ***E***, Same data as in ***B*** but using the logarithm of the animal's movement speed, with a linear function fitted to the data (red). ***F***, Z-scores of Δ*F*/*F* of cholinergic activity across test sessions (red, 10 sessions) and sample sessions (blue, 10 sessions) as a function of the logarithm of the animal's movement speed. Speed is binned with a bin width of 0.2. Data shown as mean ± SEM. ***G***, Mean ± SEM of time scale-dependent Pearson's correlation coefficient distributions between the smoothed cholinergic activity and the logarithm of the animal's movement speed (20 sessions). ***H, I***, Time-series data on movement speed and cholinergic activity, quantified as Δ*F*/*F*, from one example session (***H***) and averaged across all sessions (***I***). Time-series data are smoothed with a 1 s window for better illustration. Thick curves show the exponential fit to the data; *τ* = time constant of exponential fit. Data in ***I*** shown as mean ± SEM; the red bar shows the length of the significant cluster (zero to 18.86 s); *p = 0.002, cluster-based permutation test; *n* = 20 sessions (10 sample and 10 test sessions).

Previous experiments showed a strong correlation of cholinergic activity to the logarithm of an animal's movement speed during free foraging (Kopsick et al., 2022). We therefore first asked whether the correlation of cholinergic activity to the logarithm of the animal's movement speed was altered by the engagement of the mice in the ObLoM task. As described previously (Kopsick et al., 2022), we computed the movement speed of the animal's position using DeepLabCut and computed Pearson's correlation coefficient. As during free foraging, cholinergic activity in mice performing the ObLoM task showed a strong linear correlation to the logarithm of the animal's movement speed (Fig. 2*D*–*F*), with striking similarity to the observed relationship of cholinergic activity to the animal's movement speed found in *ad libitum* ambulating mice (Kopsick et al., 2022). The correlation between cholinergic activity and the logarithm of movement speed was significantly higher than the correlation between cholinergic activity with the movement speed (*R*_Speed_ = 0.32 ± 0.15; *R*_Log2(speed)_ = 0.38 ± 0.18; mean ± SD; *t*_(19)_ = −7.72; *p* < 0.001; paired *t* test; Fig. 2*A*,*D*), and no significant difference was found between the sample and test phases of the ObLoM task (Fig. 2*F*; Table 3).

**Table 3.** Results of an LMM predicting cholinergic activity (Δ*F*/*F*) as a function of behavioral states and movement speed

Name	Estimate	SE	*t* statistic	DF	*p* value	95% CI
Intercept	−0.487	0.099	−4.927	537,008	<0.001	[−0.68 −0.293]
Phase	0.129	0.114	1.132	537,008	0.258	[−0.094 0.351]
log₂(speed)	0.236	0.035	6.649	537,008	<0.001	[0.166 0.305]
Grooming	−0.746	0.157	−4.762	537,008	<0.001	[−1.053 −0.439]
Rearing	0.135	0.084	1.612	537,008	0.107	[−0.029 0.3]
Exploring nonstat object	0.148	0.162	0.915	537,008	0.36	[−0.169 0.465]
Exploring stat object	0.181	0.164	1.102	537,008	0.27	[−0.141 0.504]
Phase * log₂(speed)	−0.042	0.034	−1.265	537,008	0.206	[−0.108 0.023]
Phase * grooming	0.067	0.088	0.763	537,008	0.445	[−0.106 0.241]
Phase * rearing	−0.123	0.061	−2	537,008	0.045	[−0.243 −0.002]
Phase * exploring nonstat object	0.137	0.14	0.98	537,008	0.327	[−0.137 0.412]
Phase * exploring stat object	−0.158	0.202	−0.784	537,008	0.433	[−0.555 0.238]

Model formula:

$\begin{array}{r} \Delta F/F_{\mathrm{imbklt}}=\beta_{0}+\sum_{m=1}^{1}\;\beta_{1m}I[T{]}_{im+}\beta_{2}\mathrm{lo}g_{2}(\mathrm{spee}d_{i})+\sum_{b=1}^{4}\;\beta_{3b}I[B{]}_{ib}+\sum_{m=1}^{1}\;\beta_{4m}\log_{2}\;(\mathrm{spee}d_{i})I[T{]}_{im}+\sum_{m=1}^{1}\;\sum_{b=1}^{4}\;\beta_{5mb}I[T{]}_{im}I[B{]}_{ib+}b_{0k}+b_{0l}+b_{0kt}+\sum_{v=1}^{12}\;b_{vk}X_{ivk}+\sum_{v=1}^{12}\;b_{vl}X_{ivl}+\sum_{v=1}^{12}\;b_{vkt}X_{ivkt}+\varepsilon_{\mathrm{imbklt}} \end{array}$

Code for model formula:

$\begin{array}{rl} & \Delta F/F∼\mathrm{phase}\;*\;(\mathrm{speed}+\exp\_\mathrm{non}\_\mathrm{statobj}+\exp\_\mathrm{statobj}+\mathrm{rearing}+\mathrm{grooming})+(1+\mathrm{phase}\;*\;(\mathrm{speed}+\exp\_\mathrm{non}\_\mathrm{statobj}+\exp\_\mathrm{statobj}+\mathrm{rearing}+\mathrm{grooming})|\mathrm{mouse}\_\mathrm{id})\\ & \;+(1+\mathrm{phase}\;*\;(\mathrm{speed}+\exp\_\mathrm{non}\_\mathrm{statobj}+\exp\_\mathrm{statobj}+\mathrm{rearing}+\mathrm{grooming})|\mathrm{mouse}\_\mathrm{id}:\;\mathrm{ObLoM}\_\mathrm{id})+(1+\mathrm{phase}\;*\;(\mathrm{speed}+\exp\_\mathrm{non}\_\mathrm{statobj}+\exp\_\mathrm{statobj}+\mathrm{rearing}+\mathrm{grooming})|\mathrm{session}\_\mathrm{id}) \end{array}$

The response variable Δ*F*/*F*_ibmklt_ represents the *z*-scored cholinergic activity measured at observation *i*, indexed by task phase *m* (sample vs test), behavior *b* (rearing, grooming, exploring stationary object, and exploring nonstationary object), mouse *k*, session *l*, and ObLoM *t* (1 or 2, nested within mouse). Fixed effects (β) included task phase, the logarithm of movement speed, behavioral predictors, and their interaction terms with task phase (sample or test). Behavioral variables were encoded using indicator variables [*B*], and task phase using [*T*]. Random effects (b) were specified for mouse ID (*n* = 6), session ID (*n* = 20), and ObLoM ID nested within mouse ID. Random slopes were included for each fixed-effects term (*X*_v_) across these levels.

A total number of observations: 537,020; fixed-effects coefficients: 12; random-effects coefficients: 432; covariance parameters: 235. Model fit statistics, AIC = 1.3788 × 10^6^; BIC = 1.3816 × 10^6^; log-likelihood = −6.8916 × 10^5^; deviance = 1.3783 × 10^6^.

Next, we asked what the optimal timescale is for cholinergic activity to encode movement speed. After applying smoothing window sizes ranging from 0.25 to 256 s to the cholinergic signal (see Materials and Methods), we observed that the correlation between the logarithm of the animal's movement speed and the cholinergic activity was maximal at a timescale of 2.37 s (Fig. 2*G*), similar to what have been previously observed with respect to a speed code by the firing rate of MEC neurons during free foraging behavior (Dannenberg et al., 2019). Taken together, these data suggest that the animal's movement speed is robustly encoded by cholinergic activity in the septo-hippocampal system, despite the engagement of animals in a spatial memory task.

### Exponentially decaying cholinergic activity signals recency of environmental change

In addition to encoding movement speed, cholinergic activity in the basal forebrain is critically involved in regulating arousal (Marrosu et al., 1995; Acquas et al., 1996; Dannenberg et al., 2016) and spatial novelty (Aloisi et al., 1997; Giovannini et al., 2001; Bianchi et al., 2003). However, whether novelty- and arousal-triggered increases in cholinergic activity are tonic, phasic, or multiplexed remains unknown. To address this question, we analyzed fiber photometry data collected at a subsecond temporal resolution when the animal is moved by the experimenter from its home cage to the experimental arena. When the animal was placed in the arena, we observed an instantaneous increase in cholinergic activity in both the sample and test sessions, followed by a slow decay over many seconds (Fig. 2*H*, example session; Fig. 2*I*, average across all sessions).

Since cholinergic activity increases with the animal's movement speed, we asked whether the sharp increase followed by a slow decay in cholinergic activity does in fact signal the recency of a change in the environment or is simply due to increased movement speed at the beginning of the sessions. To answer this question, we fitted exponential decay functions to both the movement speed signal and the observed cholinergic activity (Fig. 2*H*,*I*) to quantify and compare their decay constants τ. While both movement speed and cholinergic activity were elevated during the initial minutes of each session, the decay time for movement speed was longer than the decay time for cholinergic activity (*τ* = 45 s vs *τ* = 28 s, respectively; Fig. 2*I*), suggesting that movement speed alone cannot account for the observed dynamics of cholinergic activity.

To further corroborate that the elevated cholinergic activity at the beginning of the sample and test sessions codes for recency of environmental change, we measured the predicted cholinergic activity using the logarithm of movement speed through linear regression (Fig. 2*H*,*I*). We then adjusted the observed cholinergic activity by removing the speed-predicted component to account for the effect of movement speed. The resulting speed-adjusted cholinergic activity remained substantially elevated at the start of the sample and test sessions, with an average decay time of *τ* = 23 s (Fig. 2*I*, bottom row). Using a nonparametric cluster-based permutation test, we confirmed that observed cholinergic activity was significantly higher than speed-predicted cholinergic activity only during the first 18.86 s of the sessions, both in terms of cluster mass (cluster mass, 2,297; *p* = 0.002; *n* = 20 sessions) and cluster length (cluster length, 18.86 s; *p* = 0.002; *n* = 20 sessions). These data suggest that cholinergic activity encodes both movement speed and recency of environmental change in parallel at different timescales.

### Mice learn and recall the location of objects in the ObLoM task

**Figure 3.**
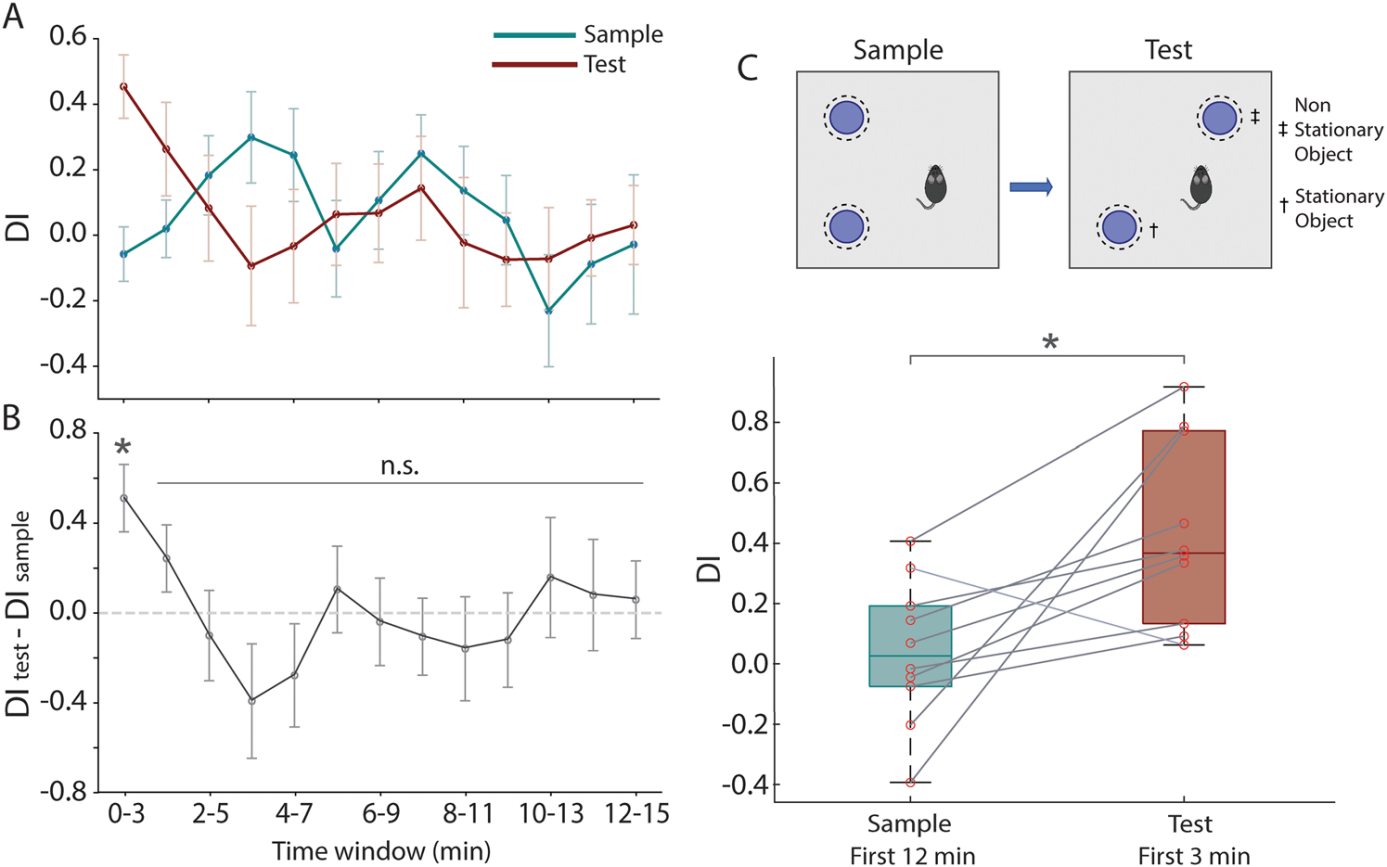
Mice explore the novel location of a nonstationary object in the ObLoM task. ***A***, DI for sample (blue) and test (red) sessions computed for 3 min intervals using a sliding window. Data show mean ± SEM. ***B***, Difference in DI between test and sample sessions computed for 3 min intervals using a sliding window; *p = 0.008; n.s., not significant; *n* = 10 sessions. ***C***, Top, Illustration of the ObLoM task. Bottom, Data on DI for sample and test sessions, computed from data on the first 12 min and 3 min, respectively. *p = 0.016; *n* = 10 sessions. DI, discrimination Index.

To evaluate whether mice successfully encoded and retrieved object location memories, we quantified their exploration preferences using the DI, a standardized measure computed as the normalized difference in exploration time between objects (see Materials and Methods). We analyzed DI values within consecutive 3 min intervals for both sample and test sessions (Fig. 3*A*). The differences in DI values between the test and sample sessions were significantly different only in the first 3 min interval (DI_Test_ = 0.45 ± 0.31 vs DI_Sample_ = −0.06 ± 0.26; mean ± SD; *t*_(9)_ = 3.4; *p* = 0.008; paired *t* test). During this initial period, mice spent significantly more time exploring the object placed in the novel location than the object in the familiar location during the test session (Fig. 3*B*). Based on these findings and consistent with the literature (Vogel-Ciernia and Wood, 2014; Schlesiger et al., 2021; Ananth et al., 2025), we used data from the first 3 min of the test session to compute the DI, while using data from the first 12 min of the sample session to compute the DI under baseline conditions. During the test but not the sample session, mice consistently devoted more time to exploring the nonstationary than the stationary object (test sessions, 13.25 ± 8.31 s vs 4.79 ± 3.44 s; mean ± SD; *t*_(9)_ = 3.54; *p* = 0.006; paired *t* test; sample sessions, 53.97 ± 41.83 s vs 43.45 ± 26.09 s; mean ± SD; *t*_(9)_ = 1.47; *p* = 0.17; paired *t* test), resulting in a significant difference in the DI between the test and sample sessions (0.43 ± 0.3 vs 0.04 ± 0.24; mean ± SD; *t*_(9)_ = 2.97; *p* = 0.016; paired *t* test; Fig. 3*C*).

### Phasic cholinergic activity signals novelty of object locations and is correlated to behaviors associated with memory-guided navigation

**Figure 4.**
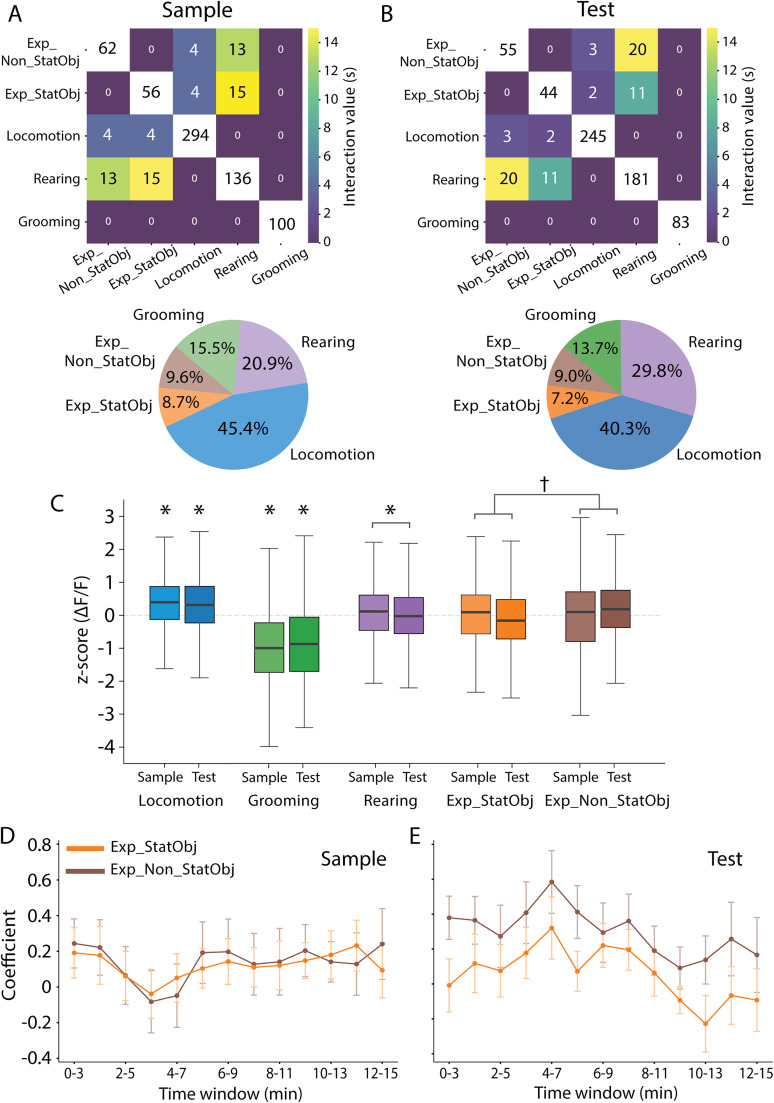
Phasic cholinergic activity signals novelty of object locations and are correlated to behavioral states. ***A***, Top, Average distribution and co-occurrence of time spent by animals in different behavioral states during sample sessions (10 sessions). Bottom, Pie charts display the proportion of behaviors during sample sessions. ***B***, Same as ***A*** but for test sessions (10 sessions). ***C***, Summary data comparing the *z*-scored cholinergic activity associated with each behavioral state during sample (10 sessions) and test sessions (10 sessions); *p < 0.001; *p* = 0.045 (rearing); ^†^*p* = 0.008 [the significance associated with locomotion refers to significance of the log_2_(speed), Table 3]. ***D***, Estimates ± SE of the LMM coefficients for exploring nonstationary and stationary objects during sample sessions (10 sessions). Coefficients of the LMM were calculated over 3 min intervals using a sliding window, including only periods with at least 1 s of object exploration. ***E***, Same as ***D*** but for test sessions (10 sessions).

We next investigated whether phasic cholinergic signals encode the novelty of an object location. Given that cholinergic activity is widely recognized as an important modulator of neural activity underpinning memory-guided navigation, we quantified cholinergic signals associated with exploring objects at familiar versus novel locations in relation to cholinergic signals associated with the logarithm of movement speed, as well as grooming and rearing. We first characterized the behavioral profiles of mice during the sample (Fig. 4*A*) and test (Fig. 4*B*) sessions. Due to the nature of these behaviors, object exploration was the only behavior that could temporally overlap with rearing or locomotion (Fig. 4*A*,*B*).

Having established the behavioral profiles, we next assessed whether cholinergic activity varied systematically across distinct behaviors. The distributions of cholinergic activity associated with each behavioral state across all sample and test sessions (Fig. 4*C*) suggested that distinct signaling patterns correspond to specific behaviors. To statistically quantify the influence of behavioral states including rearing, grooming, exploration of stationary and nonstationary objects, and the logarithm of movement speed on cholinergic activity, we employed an LMM (see Materials and Methods). In this analysis, rearing episodes included all rearing events, including rearing supported and unsupported by walls, and rearing near and away from the objects. Importantly, the model accounted for variability introduced by individual mice, sessions, and repeated tasks, including differences in baseline fluorescence and signal amplitude, thereby preserving biologically meaningful variation. Results from the LMM analysis (Table 3) revealed that the logarithm of movement speed exhibited a robust positive effect on cholinergic activity in both sample and test sessions, while grooming was associated with a negative effect in both. Although rearing did not significantly influence cholinergic activity during sample sessions, the significant negative interaction between task phase and rearing indicated that rearing was associated with lower cholinergic activity during test sessions relative to sample sessions.

We next asked whether phasic cholinergic activity specifically encodes the novelty of an object–place association. During sample sessions, both stationary and nonstationary objects were novel. During test sessions, only the nonstationary object was found at a novel location. To assess statistical significance of the difference between sample and test sessions with respect to the differential impact of exploring stationary versus nonstationary objects on cholinergic activity, we compared the object exploration-task phase interaction terms using a linear contrast analysis within the fitted LMM. The differential impact of exploring stationary versus nonstationary objects on cholinergic responses differed significantly between sample and test phases (0.137 vs −0.158; interaction terms; *F*_(1,537008)_ = 6.96; *p* = 0.008; *F* test; Table 3; Fig. 4*C*). This result suggests that phasic cholinergic activity supports the encoding of novel object–place associations. To evaluate the robustness of this effect, we performed a post hoc power analysis based on the LMM results. Using the standard deviation of the random slopes and their correlation across mice (nonstationary object, SD, 0.267; stationary object, SD, 0.412; correlation, 0.9) as input to G*Power, we computed the estimated effect size Cohen's dz = 1.43. With a two-tailed paired *t* test (*α* = 0.05) and six mice, the calculated power was 80.1%.

Our analysis of behavioral data revealed that mice exhibited a preference for exploring the object in the novel location during the first 3 min of the test session but then adopted a pattern of exploring both object locations equally (Fig. 3*A*). To determine whether this change in behavioral preference over time was aligned with changes in cholinergic activity, we applied the same LMM used above to 3 min sliding intervals of the same data (Fig. 4*D*,*E*). We then used an ordinary least squares (OLS) regression to assess how the difference between the effects of exploring nonstationary versus stationary objects on cholinergic activity varied across task phases and time windows (Table 4). During the test phase, the difference was significantly greater than during the sample phase (*β* = 0.242; SE = 0.065; *t*_(25)_ = 3.748; *p* = 0.001; Table 4; Fig. 4*D*,*E*). No significant effect of time window or interaction with task phase was observed (Table 4). These findings suggest that the spatial novelty signal by cholinergic activity remains stable throughout the task, even when differences in behavioral patterns are no longer evident.

**Table 4.** Results of an OLS regression model

Name	Estimate	SE	*t* statistic	*p* value	95% CI
Intercept	0.011	0.046	0.242	0.811	[−0.084 0.106]
Phase	0.242	0.065	3.748	0.001	[0.108 0.376]
time_window	0.001	0.006	0.107	0.915	[−0.013 0.014]
Phase: time_window	−0.002	0.009	−0.256	0.801	[−0.021 0.017]

Code for model formula:

(exp_non_statobj–exp_statobj)∼phase×time_window.

The model assesses the difference between the effects of exploring the nonstationary and stationary objects on cholinergic activity, computed by taking the difference between the coefficient for exploring the nonstationary object and the coefficient for exploring the stationary object, with coefficients extracted from the LMMs described in Table 3 applied to 3 min sliding intervals of the data. The model includes task phase (sample vs test), time window (0–3, …, 12–15 min), and their interaction term as predictors. The total number of observations, 26. Model fit statistics, *R*^2^ = 0.670; adjusted *R*^2^ = 0.625; *F* statistic = 14.87; *p* = 1.65 × 10^–5^; AIC = −49.40; BIC = −44.36; Log-likelihood = 28.70. Residual diagnostics (Omnibus = 0.503; Jarque–Bera = 0.609) indicated no significant deviation from normality.

### Changes in cholinergic activity before, during, and after behaviors associated with memory-guided navigation

Having established that phasic cholinergic activity codes for various behaviors and provides a novelty signal for object locations, we next quantified the event-triggered temporal profile of cholinergic activity associated with locomotion, grooming, rearing, and exploration of familiar and novel object locations. Toward that end, we aligned cholinergic signals with behavioral transitions into and out of each behavior, including exploration of objects, covering a window from 5 s before the onset to 5 s after the offset of a given behavior. Since the duration of individual behavioral bouts differed, we linearly time-warped behavioral bout durations between the onset and offset, so that cholinergic activity could be averaged across behavioral bouts, revealing the temporal profile of cholinergic dynamics associated with each behavior (total of 20 sessions, 10 sample, and 10 test sessions, from six mice). We used the logarithm of the animal's movement speed to predict cholinergic activity and compared the speed-predicted cholinergic signal to the observed cholinergic signal.

**Figure 5.**
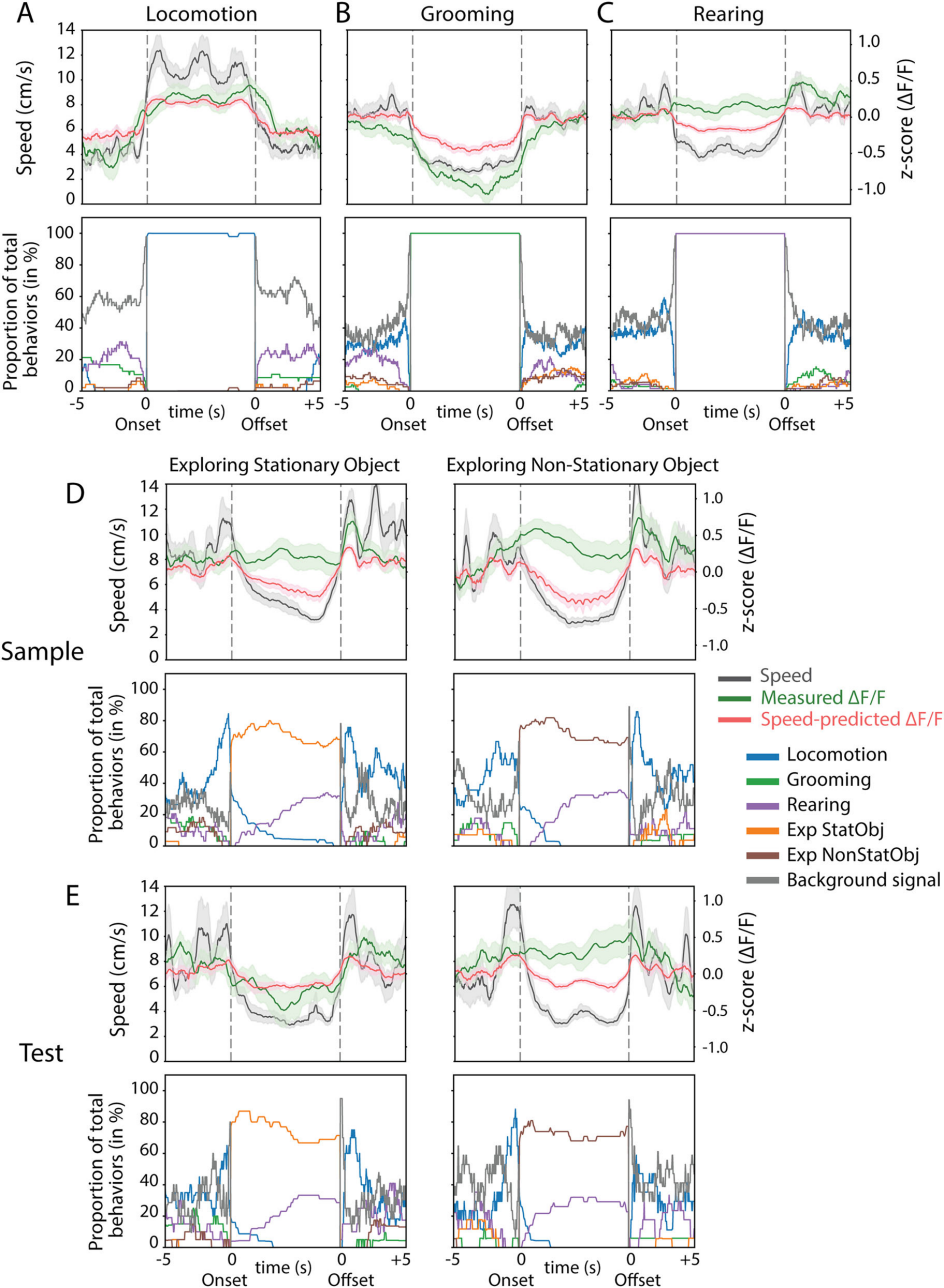
Fast cholinergic transients across cognitive and behavioral states. ***A***, Top row, Data on movement speed (black), observed cholinergic activity (green), and cholinergic activity predicted from movement speed (red) are shown for the 5 s before onset, during, and 5 s after the offset of locomotion events. Only events with no occurrence of the behavioral state in question during the 4 s before or after the onset and offset were analyzed. Between the onset and offset of a behavioral state, data are plotted on a relative timescale. Data show mean ± SEM; *n* = 47 locomotion events; data from six mice. Bottom row, Proportion of total behaviors (%) are shown for the 5 s before the onset, during, and 5 s after the offset of locomotion events. Different colors represent distinct behaviors: locomotion (blue), grooming (green), rearing (purple), exploratory behaviors associated with stationary objects (orange), exploratory behaviors associated with nonstationary objects (brown), and background (gray) indicating the absence of all other behaviors. ***B***, Same as in ***A*** for grooming. *n* = 73 grooming events. ***C***, Same as in ***A*** for rearing. *n* = 71 rearing events. ***D***, Data on exploring the stationary and nonstationary objects in the sample session. Data are visualized in the same way as in ***A***. *n* = 32 events of exploring the stationary object; *n* = 27 events of exploring the nonstationary object. ***C***, Data on exploring the stationary and nonstationary objects in the test session. Data are visualized in the same way as in ***A***. *n* = 20 events of exploring the stationary object; *n* = 17 events of exploring the nonstationary object.

Interestingly, the observed cholinergic activity increased prior to the onset of locomotion and decreased following the offset of locomotion (Fig. 5*A*). The time at which the observed cholinergic activity increased was significantly earlier than predicted from the correlation with the animal's movement speed (−0.95 ± 0.89 s vs 0.16 ± 0.81 s; median ± SD; *Z* = −2.03; *p* = 0.04; *n* = 40; Wilcoxon signed-rank test). The median time by which the increase in cholinergic signal preceded the speed-predicted increase was 1.1 s, potentially reflecting a mental preparation to move. Similarly, the decrease in observed cholinergic activity after the end of locomotion occurred later than that of the predicted signal (0.6 ± 0.67 s vs −0.08 ± 0.83 s; median ± SD; *Z* = 2.25; *p* = 0.02; *n* = 43; Wilcoxon signed-rank test), with a median delay of 0.68 s, suggesting prolonged cholinergic signaling after the end of locomotion. Despite these timing differences, there were no significant differences in the amplitude of cholinergic activity between the observed and speed-predicted signals during locomotion (cluster mass, *p* = 0.15; cluster length, *p* = 0.17; no significant cluster was identified; *n* = 47 locomotion events; cluster-based permutation test; Fig. 5*A*, top row), indicating that both signals increased similarly in magnitude. Notably, there were little to no co-occurrences of other behaviors during the analyzed locomotion events (Fig. 5*A*, bottom row).

As shown previously (Zhang et al., 2021), cholinergic activity displayed a decrease during grooming (Fig. 5*B*). In our data, this decrease was closely aligned with the predicted cholinergic signal derived from movement speed, with no significant differences at either the decrease at the grooming onset (−0.47 ± 1.07 s vs 0.13 ± 0.87 s; median ± SD; *Z* = −1.45; *p* = 0.15; *n* = 45; Wilcoxon signed-rank test) or the increase following grooming offset (0.05 ± 1.02 s vs −0.12 ± 0.93 s; median ± SD; *Z* = −1.09; *p* = 0.28; *n* = 48; Wilcoxon signed-rank test). However, the observed cholinergic activity was significantly lower than the speed-predicted activity throughout the grooming period (*p* = 0.002; 98% of grooming duration; *n* = 73 grooming events; cluster-based permutation test; Fig. 5*B*, top row), indicating a sustained suppression beyond what would be expected based on the correlation to movement speed alone. There was no co-occurrence of other behaviors during the analyzed grooming events (Fig. 5*B*, bottom row).

For rearing, cholinergic activity remained relatively constant and was consistently higher than the speed-predicted cholinergic signal across most of the rearing period (*p* < 0.03; 82% of rearing duration; *n* = 71 rearing events; cluster-based permutation test; Fig. 5*C*, top row), suggesting that cholinergic activity during rearing is influenced by factors beyond movement signals, potentially reflecting a distinct cognitive or exploratory component. There was no co-occurrence of other behaviors during the analyzed rearing events, and any instances overlapping with object exploration were excluded from the analysis (Fig. 5*C*, bottom row).

With respect to the function of cholinergic modulation in the context of providing a novelty signal for object locations, the temporal profiles revealed that cholinergic activity remained elevated relative to speed-predicted activity in all conditions with a novelty component. This included exploration of the stationary object during Sample sessions (*p* ≤ 0.014; 63% of exploration duration; *n* = 32 events; cluster-based permutation test; Fig. 5*D*, left column) and the nonstationary object during sample sessions (*p* ≤ 0.004; 89% of exploration duration; *n* = 27 events; cluster-based permutation test; Fig. 5*D*, right column), when both objects were new to the animal. Similarly, during test sessions, exploration of the nonstationary object—now placed in a novel location—also showed significantly higher observed cholinergic activity compared with the predicted signal (*p* ≤ 0.03; 61% of exploration duration; *n* = 17 events; cluster-based permutation test; Fig. 5*E*, right column). In contrast, during exploration of the stationary object at the familiar location in test sessions, cholinergic activity decreased and closely matched the predicted signal (no cluster was identified; *n* = 20 events; cluster-based permutation test; Fig. 5*E*, left column), a condition in which both the object and its location were familiar.

## Discussion

Our results demonstrate multiplexing of cholinergic signals in the septo-hippocampal system across multiple timescales. Concretely, slow changes in cholinergic activity correlate with recency of environmental changes, and phasic cholinergic activity correlates with the exploration of novel object locations, as well as movement speed and changes in cognitive and behavioral states. These data suggest that cholinergic modulation of hippocampal circuits provide multiplexed novelty signals facilitating the encoding of spatial novelty, the acquisition of spatial memories, and enabling the integration of sensory information and self-motion cues during spatial navigation.

Traditionally, cholinergic signaling was thought to be slow and diffuse, with ACh released in a tonic manner across large brain regions to regulate broad functions like wakefulness and arousal. This view emerged largely from techniques such as microdialysis (Kametani and Kawamura, 1991; Marrosu et al., 1995; Jiménez-Capdeville and Dykes, 1996). Recent research, however, has shown that phasic and tonic ACh signaling operates on both fast (subseconds to seconds) and slow (minutes to hours) timescales (Dudar et al., 1979; Dannenberg et al., 2016, 2017; Gritton et al., 2016; Záborszky et al., 2018; Disney and Higley, 2020; Sarter and Lustig, 2020; Zhu et al., 2023; Cassity et al., 2024; Yogesh and Keller, 2024), and phasic signals play a critical role in processing temporally discrete events, such as sensory stimulation and cue detection (Howe et al., 2010, 2017; Gritton et al., 2016; Zhu et al., 2023), and sensorimotor integration (Yogesh and Keller, 2024; Zou et al., 2024). Phasic signals may be facilitated by Ach release at synaptic terminals (Turrini et al., 2001; Muller et al., 2013; Takács et al., 2018) and have been implicated in the coding of movement speed, both during locomotion and in stationary mice (Kopsick et al., 2022). In contrast, tonic signals have been shown to support cognitive processes, such as learning and memory (Hasselmo, 2006; Hasselmo et al., 2017). High levels of cholinergic tone are causally linked to the “online” theta state in locomoting mice (Vandecasteele et al., 2014; Dannenberg et al., 2015) and facilitate synaptic plasticity (Záborszky et al., 2018). Conversely, low cholinergic tone during slow-wave sleep and SPW–Rs provides a permissive environment for replay (Zhang et al., 2021). However, whether septo-hippocampal cholinergic modulation uses multiplexing of fast and slow signals to support the encoding of novel spatial information, movement speed, and behaviors has remained elusive. Here, we observed a strong correlation between cholinergic activity and the brief moments during which a mouse explores a novel object location while also coding for the logarithm of movement speed. These correlations remained high at a subsecond timescale, indicating a fast or phasic mode of cholinergic signaling. At the same time, we observed an instantaneous increase followed by a slow exponential decay of cholinergic activity at each start of a session after animals transitioned from the home cage to the test arena. Taken together, these data demonstrate multiplexing of cholinergic signals and support the theory that ACh release in the hippocampus supports the encoding of spatial memories at both short and long timescales (Hasselmo et al., 1995; Hasselmo, 2006; Douchamps et al., 2013; Zhang et al., 2021).

Our data on changes in cholinergic activity during the ObLoM task demonstrate that increases in cholinergic activity associated with the exploration of a novel object location persist long after the behavioral preference for the novel object location has diminished. These data suggest that cholinergic signals may contribute to memory formation independent of changes in the animal's behavioral response. This finding is consistent with data from Thiel et al. (1998) showing that re-exposing animals to the same environment on 2 consecutive days resulted in a decrease in exploratory behavior but not in cholinergic activity levels, which were even further increased. Moreover, the data are consistent with the observation that mice fail to show a behavioral preference for a novel object location in the test phase if they are given <3 min to explore the objects in the sample phase (Stefanko et al., 2009), indicating that the formation of a spatial memory requires a mechanism that extends the time window where mice show a behavioral preference.

Cholinergic activity is recognized as an important modulator of neural activity underpinning spatial learning and memory-guided navigation (Stancampiano et al., 1999; Easton et al., 2012; McQuiston, 2014; Dannenberg et al., 2016; Haam and Yakel, 2017; Hinman et al., 2018; Solari and Hangya, 2018). We therefore investigated the temporal dynamics of cholinergic activity preceding, during, and succeeding the behavioral states of locomotion, grooming, and rearing. In particular, grooming is associated with a brain state promoting the occurrence of SPW–Rs, which are important for recall and/or consolidation of memories (Buzsáki, 2015; Joo and Frank, 2018), and rearing is associated with a brain state that may facilitate learning about the spatial environment (Lever et al., 2006). These analyses revealed that cholinergic activity was significantly reduced during grooming in both sample and test sessions, consistent with the time-locked reduction of cholinergic activity during SPW–Rs (Zhang et al., 2021). Importantly, cholinergic activity during grooming was significantly lower than what was predicted by the correlation to the animal's movement speed, suggesting that the reduction cannot be attributed to a reduction in movement speed alone. Conversely, cholinergic activity during rearing was significantly higher than what was predicted by the animal's movement speed, consistent with findings of increased cholinergic activity during rearing episodes in the context of free exploration (Kopsick et al., 2022) and with results from a different lab demonstrating (1) increased rearing duration during optogenetic inhibition of medial septal cholinergic neurons (Cassity et al., 2024) and (2) decreased spatial memory accuracy if optogenetic inactivation of the dorsal hippocampus is selectively applied during rearing epochs (Layfield et al., 2023). The former finding was attributed by the authors to animals needing extra time to encode novel information when ACh release is reduced. Although cholinergic activity during rearing was significantly higher than predicted by the animal's movement speed, the cholinergic activity levels did not increase above the absolute levels measured before and after rearing (Fig. 5*C*), consistent with microdialysis data showing no correlation between ACh levels and the number of rearing events after habituation to an environment (Thiel et al., 1998). Notably, the event-triggered analysis of cholinergic activity provided sufficient statistical power to detect the relative positive effect of rearing events on cholinergic activity, which was missed by the LMM due to the smaller (absolute) effect size compared with grooming and movement speed. Taken together, data reported here support the encoding versus retrieval scheduling framework (Hasselmo, 2006; Easton et al., 2012) of cholinergic function.

To our surprise, we found an increase in cholinergic activity before the onset of locomotion, suggesting that cholinergic signals may modulate hippocampal circuits to support the processing of movement-related sensory information. Such a function would align well with cholinergic mechanisms observed in other cortical regions, such as signaling behavioral context transitions in the auditory cortex (Kuchibhotla et al., 2017), modulation of prefrontal cortex synchrony during cue detection (Howe et al., 2017), and correlations of cholinergic signals in the visual cortex with faster and longer treadmill running (Neyhart et al., 2024). Future research is needed to probe the mechanistic functions of cholinergic signals for sensory integration and motor tasks.

With respect to novel object exploration, cholinergic activity remained high when exploring novel objects in sample sessions and when exploring the novel object location in test sessions despite associated reductions in movement speed. Moreover, the magnitude and temporal profiles of cholinergic activities matched. In contrast, cholinergic activity decreased during exploration of the familiar object location in test sessions, closely aligning with movement speed dynamics. The experimental design controlled for potential object, place, or object–place preferences as confounding factors because (1) the objects were identical, (2) control data on object–place preferences were acquired in the sample session, and (3) we used a counterbalanced task design with respect to which of the two objects were moved to a novel location. Collectively, the data are consistent with ACh providing a novelty signal for learning an object–place association.

Although novelty often leads to arousal, arousal can also arise from factors unrelated to novelty, such as emotional valence or sensory intensity (Weierich et al., 2010; Barto et al., 2013; Tanner et al., 2024). In the present experimental design, dissociating the correlated effects of novelty and arousal on cholinergic activity was not possible. Future studies using more precise behavioral and circuit-level manipulations will be needed to tease apart distinct correlations between hippocampal cholinergic activity, arousal, and spatial novelty.

While we used ChAT-Cre mice to selectively target cholinergic neurons in the MSDB, it is important to consider that subsets of these neurons may co-transmit GABA (Takács et al., 2018) or exhibit co-expression of vGLUT3 (Case et al., 2017). There is thus a possibility that the observed changes in the activity of cholinergic projection neurons alter brain and cognitive states in the hippocampus not only via changes in ACh release but also via changes in other neurotransmitters, such as co-transmitted GABA.
